# Brain iron deposition is linked with cognitive severity in Parkinson’s disease

**DOI:** 10.1136/jnnp-2019-322042

**Published:** 2020-02-20

**Authors:** George Edward Calver Thomas, Louise Ann Leyland, Anette-Eleonore Schrag, Andrew John Lees, Julio Acosta-Cabronero, Rimona Sharon Weil

**Affiliations:** 1 Dementia Research Centre, UCL Institute of Neurology, London, UK; 2 Department of Clinical Neuroscience, UCL Institute of Neurology, London, UK; 3 Movement Disorders Consortium, University College London, London, UK; 4 Reta Lila Institute for Brain Studies, University College London, London, UK; 5 Tenoke Ltd, Cambridge, UK; 6 Wellcome Centre for Human Neuroimaging, University College London, London, UK

## Abstract

**Background:**

Dementia is common in Parkinson’s disease (PD) but measures that track cognitive change in PD are lacking. Brain tissue iron accumulates with age and co-localises with pathological proteins linked to PD dementia such as amyloid. We used quantitative susceptibility mapping (QSM) to detect changes related to cognitive change in PD.

**Methods:**

We assessed 100 patients with early-stage to mid-stage PD, and 37 age-matched controls using the Montreal Cognitive Assessment (MoCA), a validated clinical algorithm for risk of cognitive decline in PD, measures of visuoperceptual function and the Movement Disorders Society Unified Parkinson’s Disease Rating Scale part 3 (UPDRS-III). We investigated the association between these measures and QSM, an MRI technique sensitive to brain tissue iron content.

**Results:**

We found QSM increases (consistent with higher brain tissue iron content) in PD compared with controls in prefrontal cortex and putamen (p<0.05 corrected for multiple comparisons). Whole brain regression analyses within the PD group identified QSM increases covarying: (1) with lower MoCA scores in the hippocampus and thalamus, (2) with poorer visual function and with higher dementia risk scores in parietal, frontal and medial occipital cortices, (3) with higher UPDRS-III scores in the putamen (all p<0.05 corrected for multiple comparisons). In contrast, atrophy, measured using voxel-based morphometry, showed no differences between groups, or in association with clinical measures.

**Conclusions:**

Brain tissue iron, measured using QSM, can track cognitive involvement in PD. This may be useful to detect signs of early cognitive change to stratify groups for clinical trials and monitor disease progression.

## Introduction

Dementia affects up to 50% of patients with Parkinson’s disease (PD)^1^ but patients vary in the timing and severity of cognitive involvement and useful quantitative tools to track cognitive change in PD are required. PD dementia is thought to be caused by the combination of amyloid, tau and α-synuclein, but the reasons for selective vulnerability of particular brain regions in PD dementia remain unclear.^2^

Neuroimaging measures sensitive to PD cognition are important to track change in clinical trials and detect early neuroanatomical correlates of cognitive involvement. Conventional neuroimaging, which uses MRI to assess volume loss caused by neuronal cell death, is poorly sensitive in PD as cell death at a large scale occurs only at later disease stages.^3^ Techniques sensitive to brain tissue microstructure are better suited to detect brain changes linked to cognitive involvement in PD.

A potential mechanism for selective vulnerability in PD dementia is excess brain iron accumulation.^4^ Iron is ubiquitous in numerous biological processes in normal ageing as well as in neurodegeneration.^5^ Brain iron accumulation is seen with age, in part due to increased blood-brain-barrier permeability,^6^ especially affecting the basal ganglia.^7–9^ The toxic potential of excess iron lies in its ability to generate reactive oxygen species,^10^ which damage DNA,^11^ irreversibly modify proteins via highly reactive aldehydes^12^ and stimulate release of iron from storage proteins leading to generation of further reactive oxygen species.^5^ This can ultimately end in iron-mediated cell death.^13^ Excess brain iron is also important in key pathophysiological pathways specific to PD.^9^ Notably, free radical species generated through iron overload interact with α-synuclein to promote Lewy-related pathology^14^ and produce neurotoxic by-products via catalysation of dopamine oxidation reactions.^15^ Increased iron is seen in the substantia nigra at post mortem in PD^16^ and in vivo using transcranial sonography.^17^

Of key significance, brain iron co-localises with Alzheimer’s pathology, particularly amyloid and tau,^18^ which are key predictors of PD dementia.^19^ Therefore, detecting levels of brain iron could be a sensitive way to identify brain tissue already affected by the earliest processes that ultimately lead to PD dementia.^20^

Quantitative susceptibility mapping (QSM) is an emerging MRI technique which detects local variations in iron content.^21 22^ QSM is sensitive to magnetic susceptibility differences between chemical species, which are captured by the signal phase of MRI gradient echo sequences. QSM recovers local susceptibility sources giving rise to magnetic field perturbations which are increased in basal ganglia regions in PD,^20^ but has never been used across the whole brain to track cognitive changes in PD.

Outcomes relating to progression of cognitive impairment are of particular interest. Recently, risk algorithms combined clinical information to predict cognitive change over time.^23^ Visual changes are also emerging as early markers of cognitive change in PD.^24^ Whether structural brain changes are more strongly linked with clinical risk scores or visual deficits before onset of dementia is not yet known.

Here, we used QSM to measure cognitive-related changes in 100 patients with PD without dementia. We hypothesised that magnetic susceptibility values reflecting brain tissue iron would be higher (1) in mesial temporal structures in relation to poorer cognitive ability; (2) in posterior and prefrontal cortical regions in relation to higher risk of dementia, measured using algorithmic scores and finally, (3)in basal ganglia regions in relation to motor change.

## Methods

### Study subjects

We recruited 100 patients with PD within 10 years of diagnosis (age 49 to 80 years, mean=66.4, SD=7.7, 48 female) to our London centre from October 2017 to December 2018. Inclusion criteria were clinically diagnosed, early to mid-stage PD (Queen Square Brain Bank Criteria) aged 49 to 80 years. Exclusion criteria were confounding neurological or psychiatric disorders, dementia and metallic implants considered unsafe for MRI. Participants continued their usual therapy (including levodopa) for all assessments. No patients were taking cholinesterase inhibitors. In addition, we recruited 37 age-matched controls (50 to 80 years, mean=66.1, SD=9.4, 21 female) from sources including unaffected patient spouses. All participants gave written informed consent. (see table 1 for participant demographics).

**Table 1:** Demographics table for participants

Measure	Control (n=37)	Parkinson’s disease (n=100)	P value
Gender (M:F)	16:21	52:48	ns
Age (years)	66.1 (9.4)	64.5 (7.7)	ns
Years of education	17.18 (2.35)	17.04 (2.84)	ns
MoCA score (out of 30)	28.6 (1.4)	28.0 (2.0)	ns
Cats-and-Dogs score	2.10 (0.58)	1.91 (0.55)	ns
Biological motion score	14.68 (9.65)	16.46 (11.49)	ns
UPDRS-III	5.5 (4.7)	18.9 (13.3)	***
Pelli-Robson contrast sensitivity	1.81 (0.22)	1.80 (0.16)	ns
Binocular LogMAR visual acuity	−0.08 (0.23)	−0.09 (0.13)	ns
D15 hue discrimination total error score	2.22 (6.46)	2.60 (4.93)	ns
HADS depression score	1.86 (2.12)	3.78 (2.81)	ns
HADS anxiety score	3.97 (3.48)	5.94 (4.12)	*
RBDSQ score	1.84 (1.42)	4.14 (2.45)	***
Smell test (Sniffin’ Sticks)	12.43 (2.44)	7.65 (3.21)	***
Disease duration (years)	N/A	4.2 (2.5)	na
Levodopa equivalent dose (mg)	N/A	457 (258)	na
Motor deficit dominance (left, right, both)	N/A	41: 54: 5	na

Means (SDs) reported.

Sleep Behaviour Disorder Screening Questionnaire. Pelli-Robson: higher score is better contrast sensitivity. Binocular LogMAR: lower score is better visual acuity. D15: lower score is better colour discrimination.

***p<0.001; *p<0.05.

HADS, Hospital Anxiety and Depression Scale; MoCA, Montreal Cognitive Assessment; ns, not significant; RBDSQ, REM (rapid eye movement) Sleep Behaviour Disorder Screening Questionnaire; UPDRS-III, Unified Parkinson's Disease Rating Scale part 3.

### Clinical assessments

Cognition was assessed using the Montreal Cognitive Assessment (MoCA). Assessment of motor function was performed using the Movement Disorder Society Unified Parkinson’s Disease Rating Scale motor part 3 (MDS-UPDRS-III) with patients in the ‘On’ state.

Risk of cognitive decline in PD was assessed using a validated clinical algorithm that combines age, UPDRS-III, REM (rapid eye movement)-sleep behaviour disorder score, sense of smell and depression to calculate 2-year risk of cognitive decline.^23^ (Smell scores and Anxiety and Depression scores were converted using a scalar conversion).

Visual acuity was assessed using the LogMAR; colour vision using the D15 and contrast sensitivity using the Pelli-Robson chart. Visual perception was assessed using two higher-order visual tasks: (1) the Cats-and-Dogs task^25^ and (2) Biological Motion, as previously described.^26^ Patients performing worse than group median performance on both tasks were classified as low visual performers (n=34).

### Imaging protocol

MRI measurements consisting of susceptibility-weighted and T1-weighted MRI scans were performed on a Siemens Prisma-fit 3T MRI system using a 64-channel receive array coil (Siemens Healthcare, Erlangen, Germany). Susceptibility-weighted MRI signals were obtained from a 2×1-accelerated,^27^ three-dimensional (3D) flow-compensated spoiled-gradient-recalled echo sequence. Flip angle 12°; echo time, 18 ms; repetition time, 25 ms; receiver bandwidth, 110 Hz/pixel. Matrix size was 204×224×160 with 1×1×1 mm^3^ voxel resolution (scan time 5 min 41 s). T1-weighted magnetisation-prepared, 3D, rapid, gradient-echo (MP-RAGE) anatomical images were acquired with the following parameters: inversion time, 1100 ms; flip angle, 7°; echo time, 3.34 ms; echo spacing, 7.4 ms; repetition time, 2530 ms; receiver bandwidth, 200 Hz/pixel. Matrix dimensions were 256×256×176 with 1×1×1 mm^3^ voxel size and 2×1 parallel acceleration was enabled (scan time 6 min 3 s).

### Voxel-based morphometry

Segmentation, normalisation to Montreal Neurological Institute (MNI) space and tissue probability modulation were carried out in SPM12 (http://www.fil.ion.ucl.ac.uk/spm/software/spm12) with default parameters, in conjunction with the DARTEL toolbox using a Gaussian smoothing kernel of 6 mm full-width-at-half-maximum. Images were compared cross-sectionally between patients and controls, and linear regression models or t-contrasts were implemented to examine associations between voxel-wise grey matter volume and clinical parameters (MoCA, motor UPDRS-III, risk scores and visual performance). Each model included age and total intracranial volume as nuisance covariates. Statistical parametric maps were generated with false discovery rate (FDR) corrected p=0.05 as the statistical cut-off.

### QSM reconstruction

**Figure 1:**
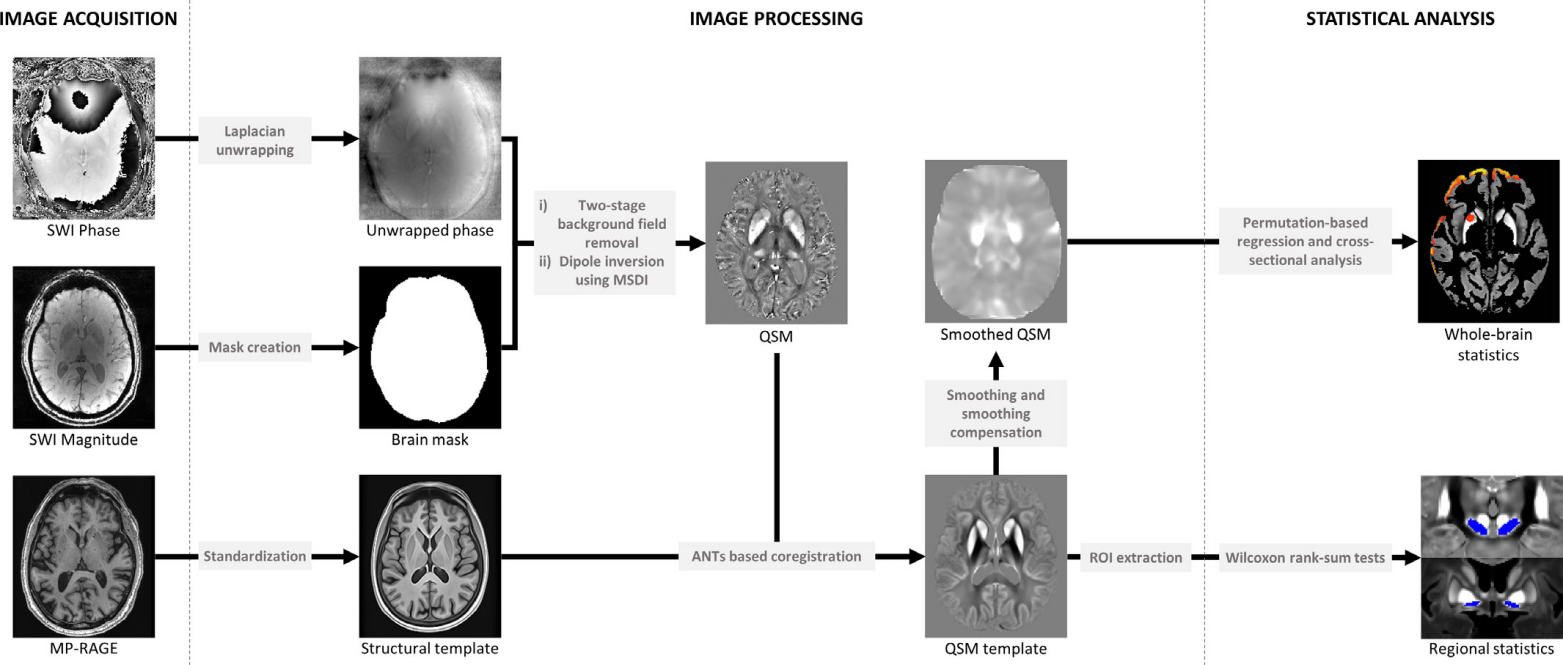
Summary steps of the processing pipeline for QSM reconstruction (phase pre-processing and map estimation) and whole brain/regional analysis. ANTs, advanced normalisation tools; MP-RAGE, magnetisation-prepared, 3D, rapid, gradient-echo; MSDI, multi-scale dipole inversion; QSM, quantitative susceptibility mapping; ROI, region of interest; SWI, susceptibility weighted imaging.

QSM image reconstruction, including phase pre-processing and estimation of susceptibility maps, followed the default QSMbox (https://gitlab.com/acostaj/QSMbox) pipeline for single-echo, coil-combined data.^28^ Three-dimensional complex phase data (adaptive combined using scanner software) were unwrapped with a discrete Laplacian method. Brain masks, which are required to separate local from background fields, were calculated (from magnitude data) using the BET2 algorithm in FSL V.5.0. (https://fsl.fmrib.ox.ac.uk). Phase pre-processing was completed in two background field suppression steps: Laplacian boundary value extraction, followed by variable spherical mean-value filtering. Susceptibility maps were estimated using a recently validated QSM algorithm, Multi-Scale Dipole Inversion,^28^ which is more robust than the previous non-linear Morphology-Enabled Dipole Inversion approach^28^ (figure 1). To increase cortical sensitivity, filtering during reconstruction was performed using a kernel with 8 mm radius.

### QSM spatial standardisation

QSM spatial normalisation and whole-brain and regional analyses were performed using QSMexplorer (https://gitlab.com/acostaj/QSMexplorer).^8^ For template creation, radiofrequency bias-corrected MP-RAGE images were spatially normalised using a previously optimised advanced normalisation tools (ANTs) (http://stnava.githib.io/ANTs) routine. In addition, bias-corrected magnitude gradient echo images were affine co-registered to their corresponding MP-RAGE volume using ANTs. QSM spatial standardisation was achieved through warp composition of the above transformations and high order interpolation. Average MP-RAGE and QSM templates were then calculated as the voxel-wise mean across subjects in study-wise space (figure 1, online supplementary figure 1).

10.1136/jnnp-2019-322042.supp1 Supplementary data

### Whole-brain QSM statistical analyses

Whole-brain analyses were performed for absolute QSM data to improve statistical conditioning in cortical regions.^29^ To attenuate the impact of misregistration and other inaccuracies, images were spatially smoothed using a 3D Gaussian kernel (3 mm SD), and were subsequently smoothing compensated^29^ using grey matter only to further improve QSM measurement specificity in cortical regions (figure 1). Probabilistic tissue segments were obtained from T1-weighted anatomical data using SPM12 (http://www.ﬁl.ion.ucl.ac.uk/spm/software/spm12). Finally, permutation analyses were performed with Randomise V.2.9 and threshold-free cluster enhancement (http://fsl.fmrib.ox.ac.uk/fsl/fslwiki/Randomise) in FSL. Significant clusters in the grey matter segment were inferred from a random subset of 10 000 data permutations and reported at FDR-corrected p<0.05. To validate methods for grey matter QSM statistical inference, a group analysis was first carried out in controls to test the hypothesis that absolute QSM covaries with age, which revealed widespread positive age effects consistent with previous ageing studies.^8^ All subsequent analyses were thus adjusted for age. We performed a cross-sectional analysis to test whether age-adjusted QSM mean values differed between PD patients and controls. Experimental analyses in the PD group were: (1) permutation based whole-brain QSM regression against MoCA and (2) against dementia risk score, (3) cross-sectional analysis of poor versus normal visual performers and(4) permutation based whole-brain QSM regression against UPDRS-III. After analysis, the QSM template and statistical maps were transformed into MNI152 space (Montreal Neurological Institute, McGill University, Canada) using a previously optimised co-registration approach.^20^

### Regional QSM statistical analyses

Several grey matter structures including the substantia nigra are typically misclassified as white matter by SPM and were not included in our whole-brain grey matter analysis. However, increased QSM would be expected in the substantia nigra in PD.^20 22^ Thus, unsmoothed, median (signed) QSM values were extracted bilaterally from the substantia nigra. The substantia nigra was manually traced in 3D from the MP-RAGE template, using FSL’s image viewer. As a post-hoc analysis, we also extracted data from the substantia innominata from the QSM template. The iron-rich substantia innominata is visually traceable on the QSM contrast, with the crossing of the anterior commissure serving as a landmark. QSM values did not differ between left/right substantia nigra or innominata segments in patients or controls (Wilcoxon rank-sum tests, both p>0.1). To improve measurement stability, median region of interest (ROI) values were averaged across hemispheres. Prior to statistical analysis, QSM values were age-corrected using the covariance method.

### Computing platform

Except where stated otherwise, processing tasks (including QSM reconstruction and regional data analysis) were executed in the MATLAB (R2012a) environment (Mathworks Inc, Natick, Massachusetts, USA). Software for QSM reconstruction and analysis are available from the QSMbox (https://gitlab.com/acostaj/QSMbox) and QSMexplorer repositories (https://gitlab.com/acostaj/QSMexplorer).

## Results

### Clinical features

One hundred and thirty-seven participants were included. One hundred patients with PD (disease duration 4.2±2.5 years), 54 patients had right predominant motor signs and 5 showed bilateral signs, plus 37 age-matched unaffected controls. Thirty-four patients were low visual performers (tables 1 and 2).

**Table 2:** Demographics table for poor and normal visual performers

Measure	Poor vision (n=34)	Normal vision (n=63)	P value
Gender (M:F)	20:14	29:34	ns
Age (years)	67.97 (7.18)	62.27 (7.20)	***
Years of education	17.99 (2.74)	16.44 (2.75)	**
MOCA score (max. 30)	27.27 (2.35)	28.37 (1.68)	**
UPDRS-III	24.59 (13.83)	21.18 (10.13)	ns
Pelli-Robson contrast sensitivity	1.70 (.16)	1.85 (.14)	***
Binocular LogMAR visual acuity	−0.05 (.14)	−0.11 (.12)	ns
D15 hue discrimination total error score	1.45 (3.99)	3.26 (5.32)	ns
HADS depression score	4.94 (3.33)	3.27 (2.31)	ns
HADS anxiety score	6.06 (4.15)	6.02 (4.16)	ns
RBDSQ score	4.03 (2.11)	4.19 (2.64)	ns
Smell test (Sniffin’ Sticks)	8.44 (3.14)	7.32 (3.24)	ns
Disease duration (years)	4.85 (2.91)	3.71 (2.16)	*
Levodopa equivalent dose (mg)	516.15 (222.09)	422.78 (272.84)	ns

Pelli-Robson: higher score is better contrast sensitivity. Binocular LogMAR: lower score is better visual acuity. D15: lower score is better colour discrimination.

***p<0.001; **p<0.01; *p<0.05.

HADS, Hospital Anxiety and Depression Scale; MoCA, Montreal Cognitive Assessment; ns, not significant; RBDSQ, REM (rapid eye movement) Sleep Behaviour Disorder Screening Questionnaire; UPDRS, Unified Parkinson's Disease Rating Scale.

### Atrophy measures

Voxel-based morphometry (VBM) showed no difference in atrophy between patients and controls, or any association with disease severity measured using cognitive, motor scores, risk score for cognitive decline or visual performance, at whole brain FDR-corrected p<0.05.

### Increased brain iron in PD compared with controls

**Figure 2:**
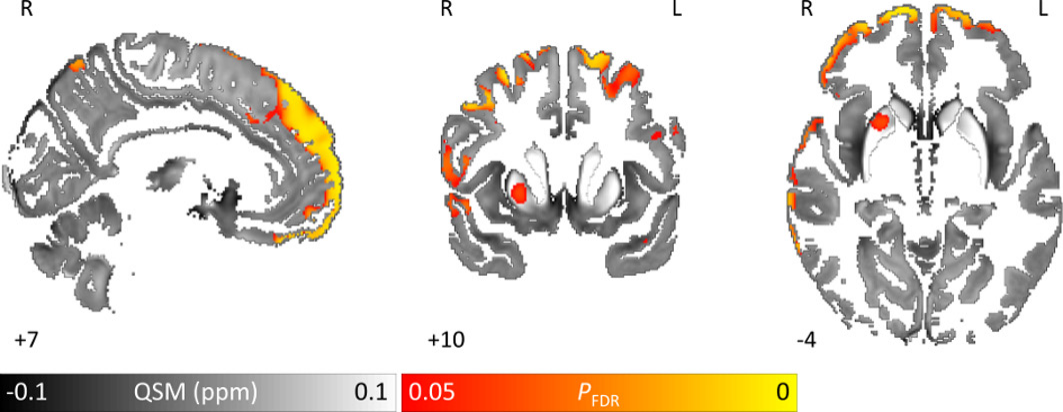
Brain tissue iron is greater in Parkinson’s disease patients compared with controls whole-brain results are overlaid onto the study-wise QSM template in the MNI coordinate system. Red/yellow clusters represent statistical differences at P_FDR_ <0.05 as indicated by the colour bar. Numbers represent position of slice in millimetres in MNI coordinate space. FDR, false discovery rate; MNI, Montreal Neurological Institute; QSM, quantitative susceptibility mapping.

Recapitulating our previous findings,^20^ we found widespread increases in QSM (FDR-corrected p<0.05) in PD relative to controls bilaterally along the cortical ribbon in prefrontal cortex (figure 2). We found unilateral QSM increases in right rostral putamen and right temporal cortex. The opposite contrast (reduced absolute susceptibility in PD) was not significant at whole-brain level.

**Figure 3:**
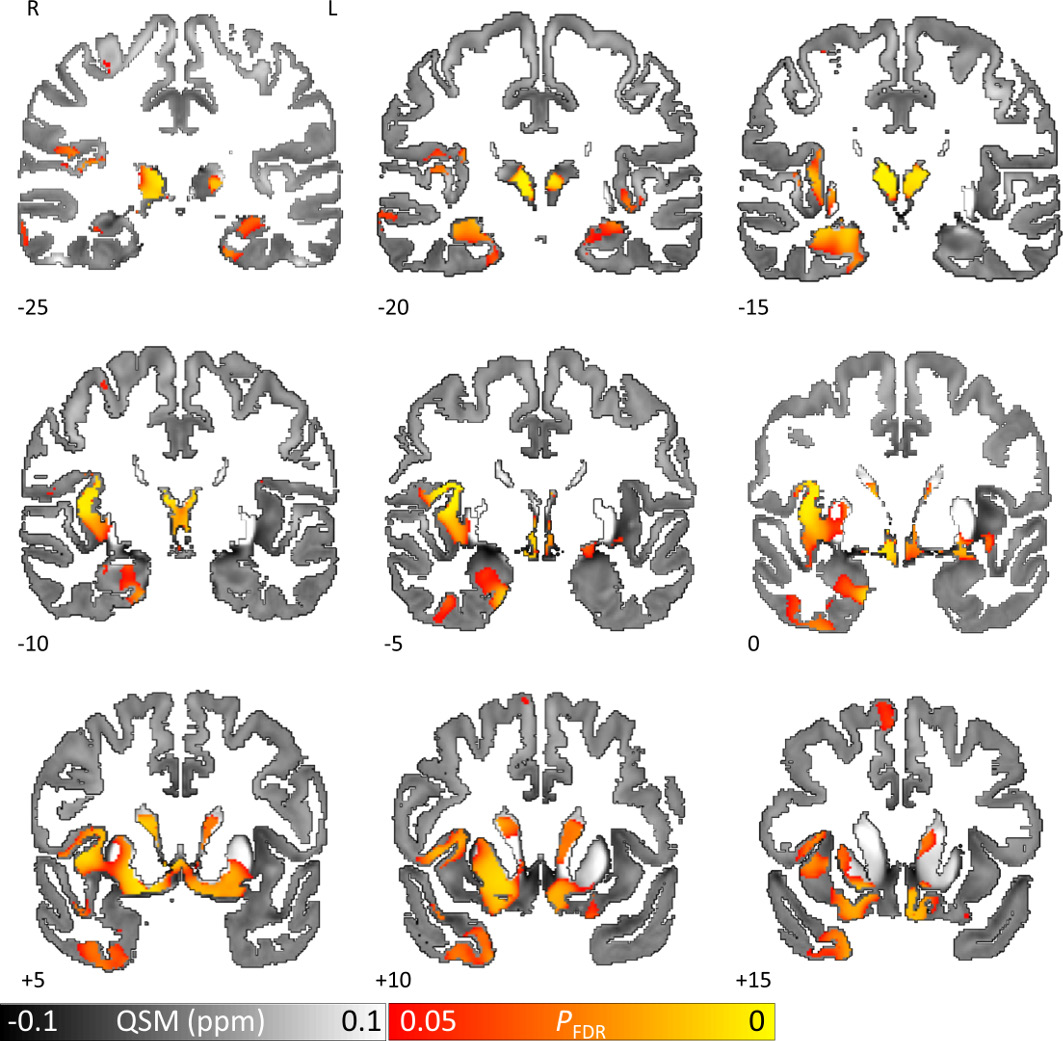
QSM covarying with lower cognitive performance (MoCA) reveals increased brain tissue iron in hippocampus, thalamus, putamen and caudate nucleus in Parkinson’s disease. Whole brain results are overlaid onto the study-wise QSM template in the MNI coordinate system. Red/yellow clusters represent statistical significance at P_FDR_ <0.05 as indicated by the colour bar. Numbers represent slice position in millimetres in MNI coordinate space. FDR, false discovery rate; MNI, Montreal Neurological Institute; MoCA, Montreal Cognitive Assessment; QSM, quantitative susceptibility mapping.

ROI analysis of the substantia nigra revealed increased (signed) QSM values (p=0.004) in PD (mean=0.092± SD=0.020) compared with controls (mean=0.082±SD=0.019), consistent with iron deposition in substantia nigra, as previously reported.^20^

### Brain iron correlates with cognition in PD

QSM regression analysis with cognition (measured using the MoCA) in patients with PD revealed bilaterally increased (FDR-corrected p<0.05) absolute susceptibility with decreasing MoCA in hippocampus, thalamus (including anterior, mediodorsal and posterior nuclei), caudal regions of ventromedial prefrontal cortex, regions of basal forebrain and rostral caudate nucleus (figure 3 online supplementary figure 2). These tissue changes, in regions known to be related to cognition, were strikingly seen where conventional measures of atrophy showed no relationship. Unilateral QSM increases with decreasing MoCA were also found in right putamen and insular cortex. We did not find increased QSM correlating with higher MoCA anywhere at whole-brain level.

10.1136/jnnp-2019-322042.supp2 Supplementary data

A post-hoc ROI analysis of the substantia innominata (to further probe iron deposition in the basal forebrain) revealed no relationship between QSM and MoCA (online supplementary figure 3).

10.1136/jnnp-2019-322042.supp3 Supplementary data

### Increased brain iron in PD with risk of rapid cognitive progression

#### Correlation with risk score

**Figure 4:**
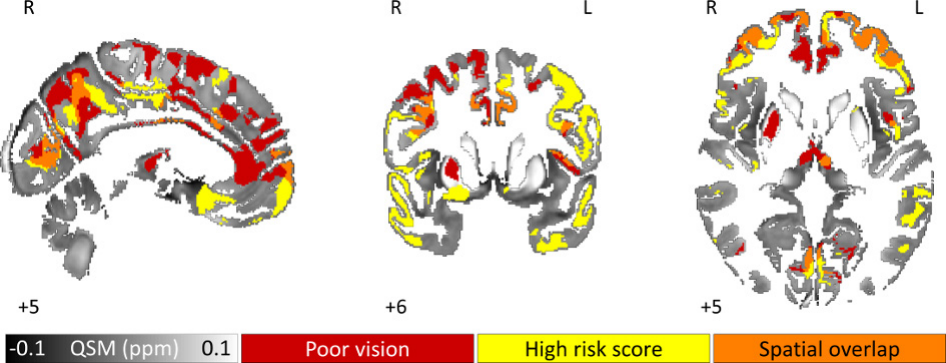
QSM greater in Parkinson’s disease at higher risk of cognitive decline as indexed by visual performance and dementia risk score. Binarised whole brain results (significant at FDR corrected p<0.05) are overlaid onto the study-wise QSM template in the MNI coordinate system. Clusters represent statistical differences for the contrasts: QSM greater in poor visual performers than in good visual performers (red) and QSM increasing with increasing dementia risk score (yellow). Orange clusters indicate the spatial overlap between these two statistical maps. Numbers represent slice position in millimetres in MNI coordinate space. FDR, false discovery rate; MNI, Montreal Neurological Institute; QSM, quantitative susceptibility mapping.

QSM regression against dementia risk score^23^ in PD patients revealed widespread increases in absolute susceptibility with increasing risk score (FDR-corrected p<0.05) (figure 4). Bilateral increases were seen in prefrontal, frontal, cingulate, temporal, parietal and medial occipital cortex as well as basal forebrain. QSM did not increase with decreasing risk score anywhere at whole-brain level.

#### Poor versus normal visual performers

Analysis of PD patients with poor versus normal visual performance revealed widespread QSM increases in patients with poor visual performance (FDR-corrected p<0.05) (figure 4). Bilaterally, increased brain iron was seen in prefrontal, frontal, cingulate, parietal and medial occipital cortex as well as mediodorsal thalamic nucleus. An increase was also seen in right putamen. The reverse contrast returned no significant clusters. There was striking overlap of brain iron levels between patients with high dementia risk and those with poor vision scores in prefrontal, anterior cingulate and posterior parietal cortex and precuneus regions, suggesting common underlying substrates (figure 4).

### Brain iron and motor scores

**Figure 5:**
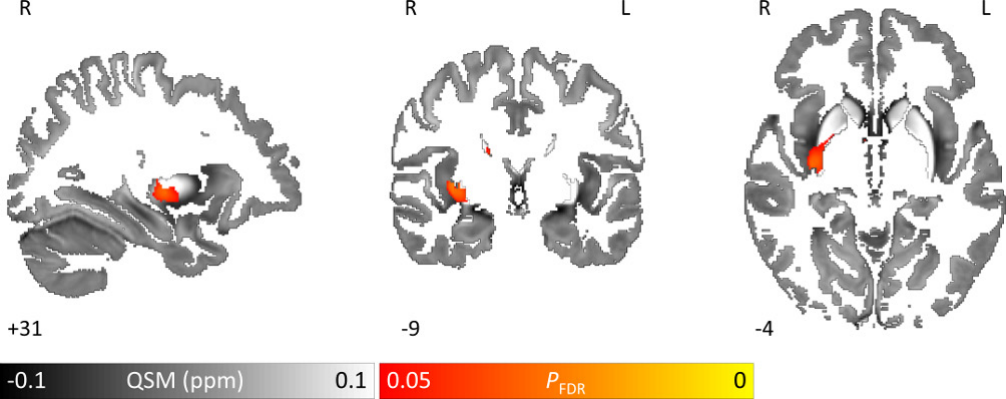
QSM covarying with higher motor scores (UPDRS-III) reveals increased brain iron in the right putamen in Parkinson’s disease. Whole brain results are overlaid onto the study-wise QSM template in the MNI coordinate system. Red/yellow clusters represent statistical significance at P_FDR_ <0.05 as indicated by the colour bar. Numbers represent position slice in millimetres in MNI coordinate space. FDR, false discovery rate; MNI, Montreal Neurological Institute; QSM, quantitative susceptibility mapping; UPDRS-III, Unified Parkinson’s Disease Rating Scale part 3.

QSM regression analysis against UPDRS-III score within the PD group revealed a significant increase (FDR-corrected p<0.05) with UPDRS-III in right caudal putamen, (figure 5). At lower thresholds (p=0.07), bilateral increases in brain iron were seen. The opposite contrast (QSM increasing with decreasing UPDRS-III) revealed no significant clusters.

A further exploratory ROI analysis of the substantia nigra, revealed no significant relationship between QSM and UPDRS-III.

## Discussion

We used QSM to identify brain tissue iron changes relating to poorer cognition in PD. For the first time, we showed tissue changes within hippocampus and thalamus relating to cognitive deficits in PD without dementia, and that brain iron increased in parietal and prefrontal cortices relating to predictors of poor cognitive outcome. Additionally, we showed that brain iron increased in the putamen in relation to poorer motor function. These anatomically specific changes were detected where conventional neuroimaging failed to identify atrophy and without requiring predefined regions of interest. Our findings have important potential as neuroimaging markers of disease activity with application in the clinic and in therapeutic trials.

### Tissue changes reflecting poorer cognition

We showed that cognition assessed with the MoCA correlated with magnetic susceptibility in hippocampus and thalami, as well as ventromedial prefrontal cortices, basal forebrain and rostral caudate nucleus. Previous studies using conventional structural measures report varying patterns of grey matter atrophy in patients with early cognitive change in PD^30^ with several showing no atrophy changes.^31^ Hippocampal atrophy is reported in cross-sectional studies in PD,^32^ and is the most consistently abnormal region in longitudinal structural MRI.^33^ Thalamic changes are also linked with cognition in PD.^30^ However, a significant relationship between MoCA and grey matter volume has not been shown in PD.^34^

In contrast, this study showed that cognition correlated with QSM in hippocampal regions, suggesting that QSM is more sensitive to early tissue changes than conventional atrophy measurements. Previous ROI analyses reported increased QSM in bilateral hippocampus and thalamus in PD dementia.^22^ A recent ROI analysis found increased QSM in PD-MCI (mild cognitive impairment) relative to PD in regions including precuneus and orbitofrontal cortex, and higher QSM with decreasing MoCA in cuneus and caudate nucleus.^35^ Here we show for the first time that cognitive scores relate to QSM signal detected in a whole-brain analysis. Correlations between QSM and MoCA scores were greater in right than left hippocampus. Right hippocampus is linked with spatial rather than verbal memory,^36^ consistent with observations that memory changes in PD affect spatial memory.^37^

We also found QSM increases with lower MoCA scores in the caudate nucleus. This is consistent with findings in other modalities; functional MRI caudate activation is reduced during memory tasks in PD-MCI^38^ and cognitive decline in PD is linked to lower caudate uptake in dopamine transporter single-photon emission computed tomography (DAT-SPECT) and positron emission tomography imaging.^39^ We found QSM increases in basal forebrain with lower MoCA scores, although not in the substantia innominata (online supplementary figure 3). The substantia innominata is the major source of cholinergic innervation to the cerebral cortex^40^ and shows post-mortem cell depletion and Lewy-related pathology^41^ in Parkinson’s dementia. The lack of covariance here could suggest this is a later event in Parkinson’s dementia.

Our finding of QSM changes in hippocampal regions in patients with poorer cognition may reflect increased levels of Alzheimer’s pathology in these regions. In Alzheimer’s disease, higher QSM, especially in the hippocampus, is seen in amyloid-positive patients.^18^ Iron may bind to amyloid beta, worsening its toxicity either directly^42^ or via increased phosphorylation of tau.^43^ Additional precedents for higher QSM signal in hippocampal regions are those in a beta amyloid transgenic mouse model^44^ and in a mouse model of tauopathy.^45^ Future work with post-mortem tissue can clarify the precise pathological proteins driving the magnetic susceptibility changes we found in relation to PD cognition. Either way, the ability of QSM to non-invasively detect changes related to tissue pathology using MRI (rather than PET) has important potential for tracking disease activity with applications for clinical trials in PD dementia.

### Tissue changes relating to risk of rapid cognitive progression

In addition to examining the relationship with current cognitive status, we investigated QSM changes linked to risk of PD dementia using an algorithmic risk score.^23^ Structural brain changes related to this risk score have not yet been examined. In our cohort, VBM analysis did not show any association with dementia risk. However, a recent study using a separate clinical risk score showed cortical thinning in similar regions to those shown in our QSM analysis, supporting the regions we found as implicated in early stages of cognitive change in PD.^46^

Impaired visuoperception is emerging as a potential predictor of Parkinson’s dementia. Patients with Parkinson’s dementia show occipital hypoperfusion prior to dementia onset^47^ and post-mortem studies show more rapid dementia in patients with PD with occipital Lewy-related pathology.^48^ We recently showed that poor visual performance is linked with worse cognition after 12 months^24^ and that visual tests relate to risk of dementia in PD.^26^ Our finding that prefrontal, occipital and posterior parietal regions showed increased iron content is consistent with our recent report of reduced connectivity between posterior and frontal brain regions in PD patients with poor visual function.^24^ We showed several regions of overlap (notably prefrontal and posterior parietal regions) between clinically-defined risk, and risk defined using visual performance. Although we find a negative correlation between visual performance and dementia risk scores, the visual scores do not themselves form part of the risk algorithm, thus avoiding statistical circularity. As both risk scores capture patients at higher risk of dementia, it is unsurprising to find a relationship between them and the overlap in brain regions is likely to represent a common underlying biological substrate relating to risk of dementia. Ultimately, longitudinal data will be needed to determine which of these indices is the better predictor for cognitive decline in PD.

### Tissue changes reflecting motor severity

We showed increased QSM in the PD putamen with poorer motor function. PD is characterised by loss of dopaminergic projections from the substantia nigra to the putamen,^49^ and the putamen has a strong relationship with motor function in PD at post mortem.^50^ In vivo, ^18^Fluorodopa and dopamine tracers show consistent and strong correlations between putaminal dopamine uptake and motor function.^51^ This relationship between motor severity and brain tissue iron has not been previously shown.^20^ This may be due to lack of more severe patients in earlier studies, and because patients were assessed as a group against controls, rather than across the spectrum of motor severity.

We did not find atrophy changes relating to motor severity, consistent with previous studies using conventional grey matter analyses.^52^ A recent study with a much larger number of patients (n>300) showed voxel-based correlations between grey matter and UPDRS-III in striatal regions.^53^ This suggests that VBM can detect striatal abnormalities in PD but is relatively insensitive, and that QSM provides greater sensitivity to identify disease-related changes.

QSM changes relating to motor severity were seen only on the right hemisphere at corrected thresholds, despite our cohort showing higher frequency of right-sided signs (and therefore left-brain involvement). Bilateral changes were seen at lower thresholds, but the preferential involvement of right-sided changes in the putamen may reflect asymmetrical patterns of iron deposition. Intriguingly, the role of the putamen in motor function is thought to relate more to axial symptoms including gait and posture, rather than bradykinesia and rigidity.^54^ This may explain the observation of a correlation with motor function in the ‘On’ state in the putamen, as axial symptoms are less responsive to treatment than limb symptoms.^55^ Axial changes may also not perfectly align with laterality of limb changes, which could explain our findings being to the right side.

We did not find increasing QSM in the substantia nigra (SN) with decreasing UPDRS-III at ROI level, although we did find increased QSM in the SN in PD relative to controls. As most dopaminergic SN neurons have degenerated PD diagnosis,^49^ and iron overload is thought to be an early event in neurodegeneration,^5^ it follows that high SN iron accumulation is already present in most of the PD group. Thus, differences in motor ability in established PD may be better explained by variations in QSM in other regions, such as the putamen.

Motor scores were performed during the ‘On’ state to avoid cognitive confounds of anxiety arising from being in the ‘Off’ state during cognitive testing. ‘On’ state UPDRS worsens with disease progression and is widely used in studies of PD disease progression^56^ and in the validation series of the UPDRS.^57^ Our finding of putaminal magnetic perturbations relating to ‘On’ state UPDRS reflects a measure of motor disease activity, although with additional signal relating to response to treatment.

### Brain tissue iron and selective vulnerability in Parkinson’s degeneration

Our results show that iron in the PD brain has an important relationship with clinical severity. Behavioural changes, captured by clinical measures, often occur before consistent atrophy is seen in PD.^58^ These clinical changes likely indicate dysfunctional activity from disruption of cellular function that will ultimately lead to neurodegeneration. Importantly, elevated iron can interfere with mitochondrial function,^59^ stimulate aggregation α-synuclein aggregation^14^ and compromise microglial neuroprotection,^60^ all potential triggers for cellular and tissue damage. The basal ganglia, key in PD network dysfunction, accumulates iron with ageing^7 8^ and has higher iron content in PD.^20^ Thus, it is especially sensitive to effects of iron dyshomeostasis that occur early in disease progression.^9^ The reasons for selective sequestration of iron by the basal ganglia, however, are not yet known.^5 9^

### Brain iron and other dementias

Brain tissue iron has previously been examined in patients with Alzheimer’s disease, using QSM, with higher levels seen in putamen as well as in amygdala and caudate^61^ and also in hippocampus, amygdala, precuneus and thalamus.^62^ An inverse correlation was also seen with poorer cognitive performance in the caudate, in a recent region of interest analysis.^63^ The distribution of susceptibility change, particularly in relation to cognitive performance, differs from that seen in our cohort, suggesting that the relationship to cognition that we found may not be entirely explained by Alzheimer’s pathology in our cohort. Similarly, patients with vascular dementia showed higher levels of brain susceptibility in the caudate and putamen, in a region of interest analysis.^64^ In patients with subcortical vascular mild cognitive impairment, higher susceptibility values were found in bilateral hippocampus, and right putamen compared with controls, with an inverse relationship between these susceptibility values and cognitive test scores.^65^ Whether common mechanisms underlie the susceptibility changes in vascular dementia, Alzheimer’s dementia and in early stages of Parkinson’s dementia will need to be specifically examined in future studies that include histological analyses, or concurrent radio-ligand imaging.

### Limitations

To increase cortical sensitivity, susceptibility map reconstruction used a spatial filter expected to recover susceptibility sources resulting from localised dipole fields. However, this may have limited ability to recover susceptibly sources arising from larger dipole fields.

Segmentation of the whole brain into grey and white matter was performed to reduce erroneous variability along the cortical ribbon resulting from difference in grey and white matter susceptibility. However, areas such as the posterior thalamic nuclei are split by this segmentation. Widespread thalamic iron increase was seen relating to cognitive involvement and findings in the posterior region may have been distorted by this segmentation. Future work should examine varying QSM findings in thalamic subregions.

Measures shown here were obtained at a single time point. Future longitudinal studies can examine the ability of QSM to predict changes in disease course

## Conclusion

In summary, we show that whole brain measures of iron content can be used to probe key clinical indices of disease activity, with cognitive performance related to hippocampal changes, dementia risk linked to increased brain iron in parietal and frontal cortices and motor severity co-varying with raised brain iron levels in the putamen.
