# Correction: *Brain iron deposition is linked with cognitive severity in Parkinson’s disease*

**DOI:** 10.1136/jnnp-2019-322042corr1

**Published:** 2021-04-12

Thomas GEC, Leyland LA, Schrag A, et al. Brain iron deposition is linked with cognitive severity in Parkinson’s disease. *J Neurol Neuro Psychiatry* 2020;91:418-425.

Instead of p maps as inputs to generate the FDR thresholds, raw T maps were erroneously used. Where FDR-corrected whole-brain analyses at p<0.05 were presented, uncorrected thresholds are as follows: p<0.025 for QSM greater in PD than controls, p<0.025 for QSM regression against MoCA, p<0.026 for greater QSM in PD poor visual than normal visual performers, p<0.027 for QSM regression against dementia risk score and p<0.015 for QSM regression against UPDRS-III score.

