# Supplementary figures and images for: Brain iron deposition is linked with cognitive severity in Parkinson’s disease
Source: J Neurol Neurosurg Psychiatry. 2020 Feb 20;91(4):418–25. doi: 10.1136/jnnp-2019-322042 (PMC7147185; doi:10.1136/jnnp-2019-322042)

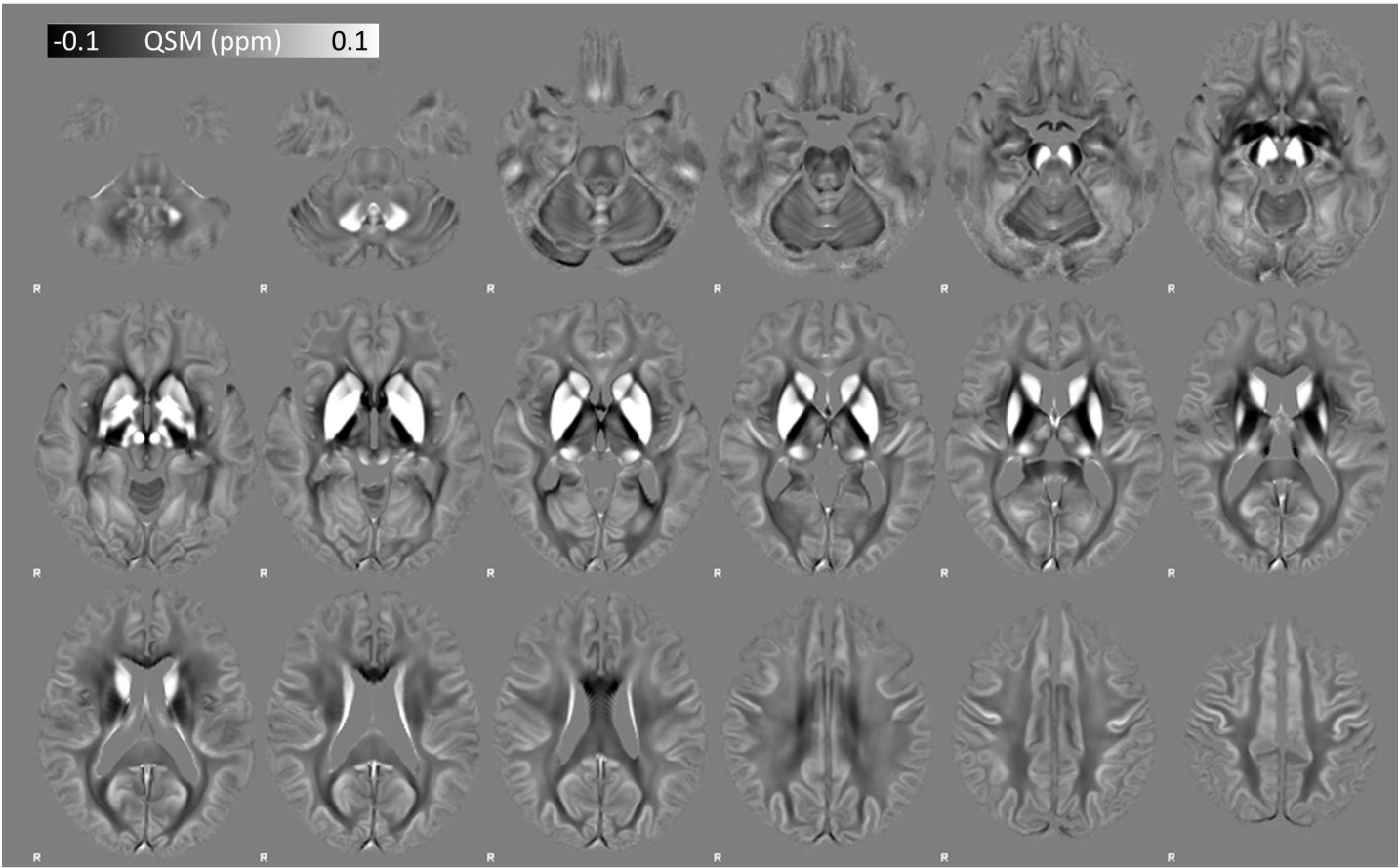

Supplement: Supplementary data [file jnnp-2019-322042supp001.pdf]

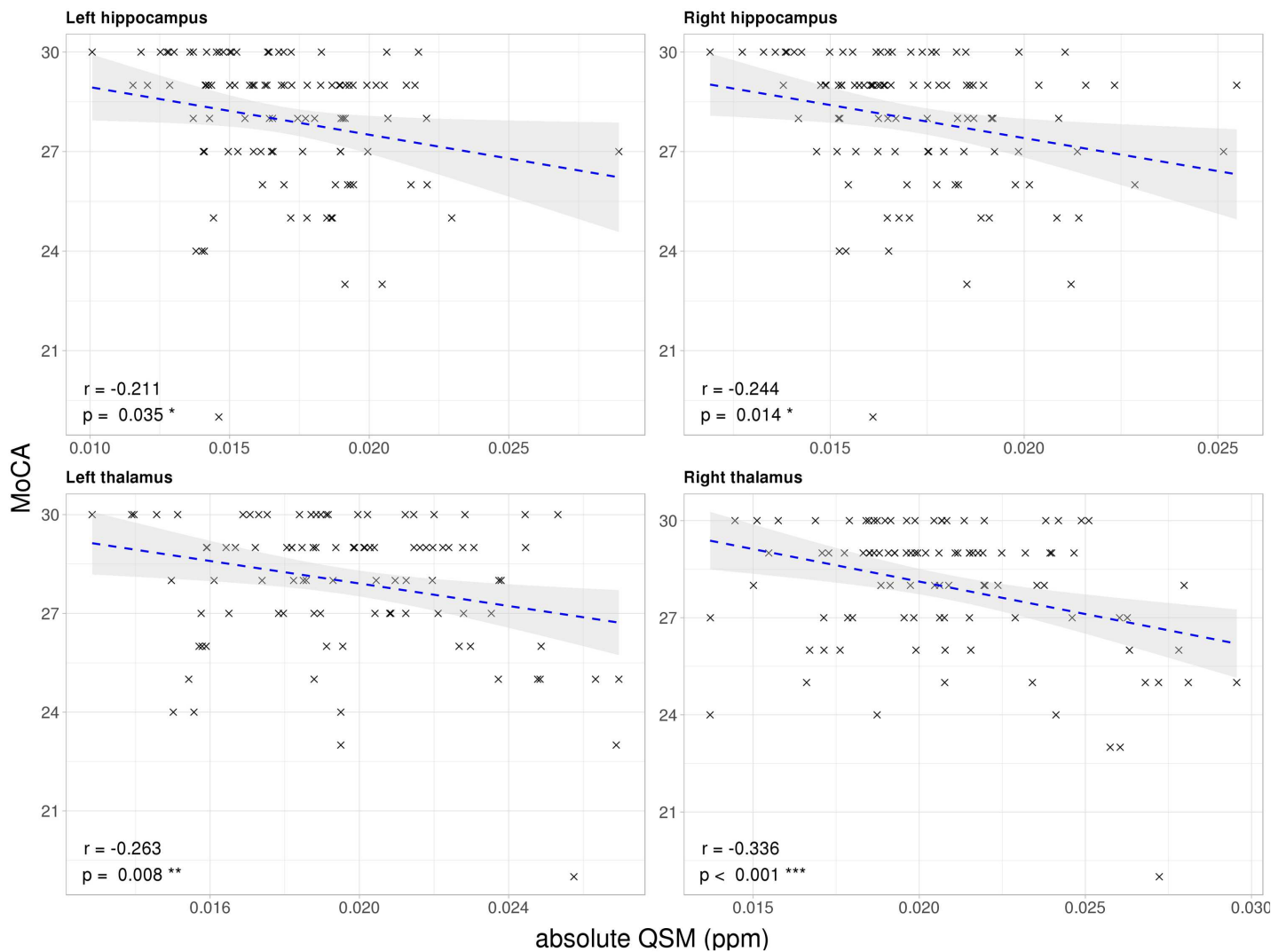

Supplement: Supplementary data [file jnnp-2019-322042supp002.pdf]

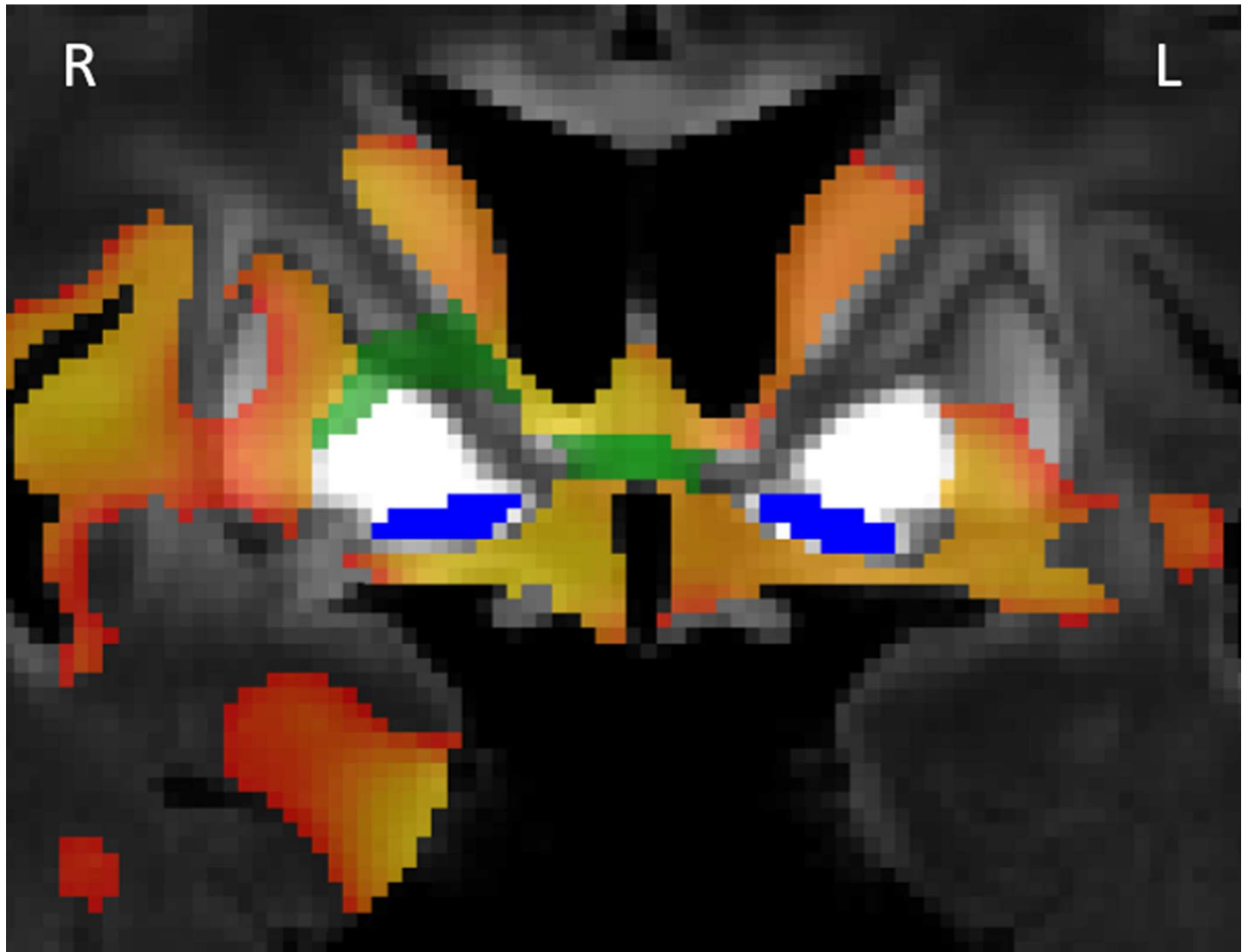

Supplement: Supplementary data [file jnnp-2019-322042supp003.pdf]
